# Biochemical characterization of the full-length isoform of soluble adenylyl cyclase

**DOI:** 10.1016/j.jbc.2025.110889

**Published:** 2025-11-05

**Authors:** Jacob Ferreira, Hayden Belliveau, Lonny R. Levin, Jochen Buck

**Affiliations:** Department of Pharmacology, Weill Cornell Medicine, New York, New York, USA

**Keywords:** soluble adenylyl cyclase (sAC), *ACDY10*, isoforms, bicarbonate, calcium, cyclic adenosine monophosphate (cAMP)

## Abstract

cAMP is a ubiquitous second messenger produced from ATP and involved in many cellular processes. In humans, cAMP is produced from two types of adenylyl cyclases: G protein–regulated transmembrane adenylyl cyclases (*ADCY1-9*) and bicarbonate- (HCO_3_^−^) and calcium- (Ca^2+^) regulated soluble adenylyl cyclase (sAC; *ADCY10*). sAC is molecularly and biochemically distinct from other mammalian nucleotidyl cyclases. In most mammals, sAC arises from a single gene which is predicted to generate multiple isoforms *via* alternative splicing. In rodents, there are two molecularly identified splice variants: the “full-length” isoform (sAC_fl_) and a “truncated” isoform (sAC_t_). To date, biochemical and structural characterization of sAC has focused almost exclusively on the sAC_t_ isoform. Longer sAC isoforms, including the longest known sAC_fl_, contain additional presumptive regulatory domains which have not yet been functionally characterized. Thus far, studies have been limited by the inability to obtain sufficient sAC_fl_ protein to allow *in vitro* biochemical characterization. Here, we describe attempts to heterologously express and purify human sAC_fl_ as well as generation of a novel genetically modified mouse strain which permits biochemical separation and purification of endogenously expressed mouse sAC_fl_ and sAC_t_. We use these heterologously expressed and endogenous proteins to compare and contrast the biochemically properties of human and mouse sAC_fl_ and sAC_t_.

cAMP is a ubiquitous second messenger involved in various intracellular signal transduction pathways throughout the bacterial and animal kingdoms. In mammals, cAMP mediates a myriad of effects *via* its numerous effectors; including protein kinase A, exchange proteins activated by cAMP, and cyclic nucleotide-gated channels (1, 2, 3). With the appreciation that cAMP can mediate various, oftentimes opposing, cellular signals within a single cell type, it became apparent that cAMP is compartmentalized into independently regulated signaling nanodomains. These nanodomains are characterized by the presence of specific adenylyl cyclases (ACs), the enzymes which produce cAMP by cyclizing ATP, a specific effector protein, and a phosphodiesterase, which are enzymes responsible for degrading the second messenger (4). Nanodomains control the spatial and temporal regulation of cAMP by preventing the spread of signals to neighboring nanodomains and regulating the duration of signaling events.

In most mammals, 10 genes encode the known AC isoforms (*ADCY1-10*). These can be categorized into two classes transmembrane adenylyl cyclases (tmACs) and soluble adenylyl cyclase (sAC). Nine genes (*ACDY1-9*) encode different tmACs, which are regulated by heterotrimeric G proteins and mediate cAMP signaling downstream from hormones and neurotransmitters modulating G protein–coupled receptors (5). A 10^th^ gene (*ADCY10*) encodes sAC (6), which lacks predicted transmembrane domains, is not subject to regulation by heterotrimeric G proteins, and localizes across various cellular compartments such as the cytosol, mitochondria, and nucleus (7, 8, 9, 10, 11, 12). sAC activity is regulated by Ca^2+^ and HCO_3_^–^ ions and is sensitive to physiologic fluctuations in ATP (13, 14, 15, 16). Though widely expressed in somatic tissues, sAC is most abundantly expressed in male germ cells (6, 17, 18). sAC-generated cAMP has numerous functions distinct from the roles played by cAMP generated by tmACs [reviewed in (19, 20, 21)]. Its most prominent function is regulating motility and post-ejaculation maturation (*i.e.*, capacitation) of sperm (22, 23, 24, 25, 26). Before ejaculation, morphologically mature but functionally dormant sperm are stored in the epididymis. Upon ejaculation, sAC activity in sperm is stimulated by an increase in HCO_3_^−^ and Ca^2+^ in seminal fluid and the female reproductive tract causing the sperm to become motile and acquire the ability to fertilize an oocyte, a phenomenon termed capacitation (27, 28, 29). Due to its control of these essential functions in sperm, humans and mice homozygous for sAC inactivating mutations are male-specific sterile (22, 24, 26, 30), and sAC is actively being pursed as a potential target for novel nonhormonal contraceptives (25, 31, 32).

Upon initial purification and molecular cloning of rat sAC (6), two discrete cDNAs were isolated which arose *via* alternative splicing from the single *ADCY10* locus (33). The first transcript represents a full-length sAC isoform (sAC_fl_) encoded by all 33 exons in the ADCY10 gene and encoding a 187 kDa protein. sAC_fl_ contains two heterologous catalytic domains (C1 and C2) and is predicted to contain multiple C-terminal regulatory domains including an autoinhibitory domain (34), a phosphate-binding loop (P-loop) consensus sequence (35, 36), and a predicted heme binding domain (37) (Fig. 1). The second transcript skips the 12th exon of the *ADCY10* gene and introduces a premature stop codon, generating a truncated sAC isoform (sAC_t_) of 53 kDa which corresponds to the protein originally purified from rat testes (6, 33). The absence of exon 12 in the sAC_t_ encoding transcript represents a potential site for selectively targeting the different transcripts with RNA directed methods. In contrast to sAC_fl_, sAC_t_ contains only the C1 and C2 catalytic domains, and due to the absence of the presumptive autoinhibitory domain, sAC_t_ exhibits higher specific activity than sAC_fl_ (13, 34, 38). Molecular cloning and multiple genomics and transcriptomic studies predicted additional isoforms in humans and rodents, including isoforms lacking the first catalytic domain (sAC-C2 only) with as yet indeterminate catalytic activity (17, 37, 39, 40, 41).

**Figure 1:**
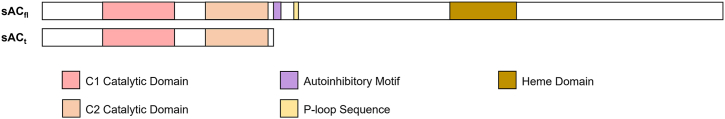
**Structural organization of sAC_fl_ and sAC_t_.** Schematic of full-length (sAC_fl_) and truncated (sAC_t_) sAC isoforms with catalytic domains and presumptive regulatory domains highlighted. sAC_fl_ is a 187 kDa protein, which contains two heterologous catalytic domains (C1 and C2) and multiple predicted C-terminal regulatory domains including an autoinhibitory domain (34), a P-loop consensus sequence (35, 36), and a predicted heme binding domain (37). Conversely, sAC_t_ skips an exon introducing a premature stop codon, which produces a 53 kDa truncated protein. P-loop, phosphate-binding loop; sAC, soluble adenylyl cyclase.

Biochemical and structural characterization of sAC has focused almost exclusively on activity of the sAC_t_ isoform, because it proved amenable to heterologous expression and purification (15, 42). sAC_fl_ has thus far resisted *in vitro* characterization because heterologous expression and attempts to purify this isoform have proved challenging (34). Thus far, *in vitro* studies have been unable to study sAC_fl_ on its own. Experiments characterizing sAC_fl_ were performed using either extracts from HEK293 cells transfected with sAC_fl_ constructs or immunoprecipitations (IPs) of endogenous sAC isoforms from testes (13, 34, 38, 43), and both cases, activities measured would reflect contributions from tmACs or sAC isoforms other than sAC_fl_.

Given the pivotal role of sAC in sperm function, there is significant interest in developing sAC inhibitors as novel, on-demand, nonhormonal male, and/or vaginally delivered female contraceptives (25, 31, 32). A potential impediment to these efforts is a clear appreciation of the specific contributions of different sAC isoforms to the various roles played by sAC-generated cAMP in sperm and throughout the body. For example, if individual sAC isoforms uniquely contribute to essential sperm functions, they could be targeted as contraceptives by isoform-specific reagents which would diminish unwanted adverse effects. To improve our understanding of sAC biochemistry and identify whether there are biochemical or pharmacological distinctions between sAC_fl_ and sAC_t_ which might be leveraged for therapeutic benefit, we sought to isolate and independently characterize the sAC_fl_ isoform.

## Results

### Novel method to heterologously express sAC_fl_

**Figure 2:**
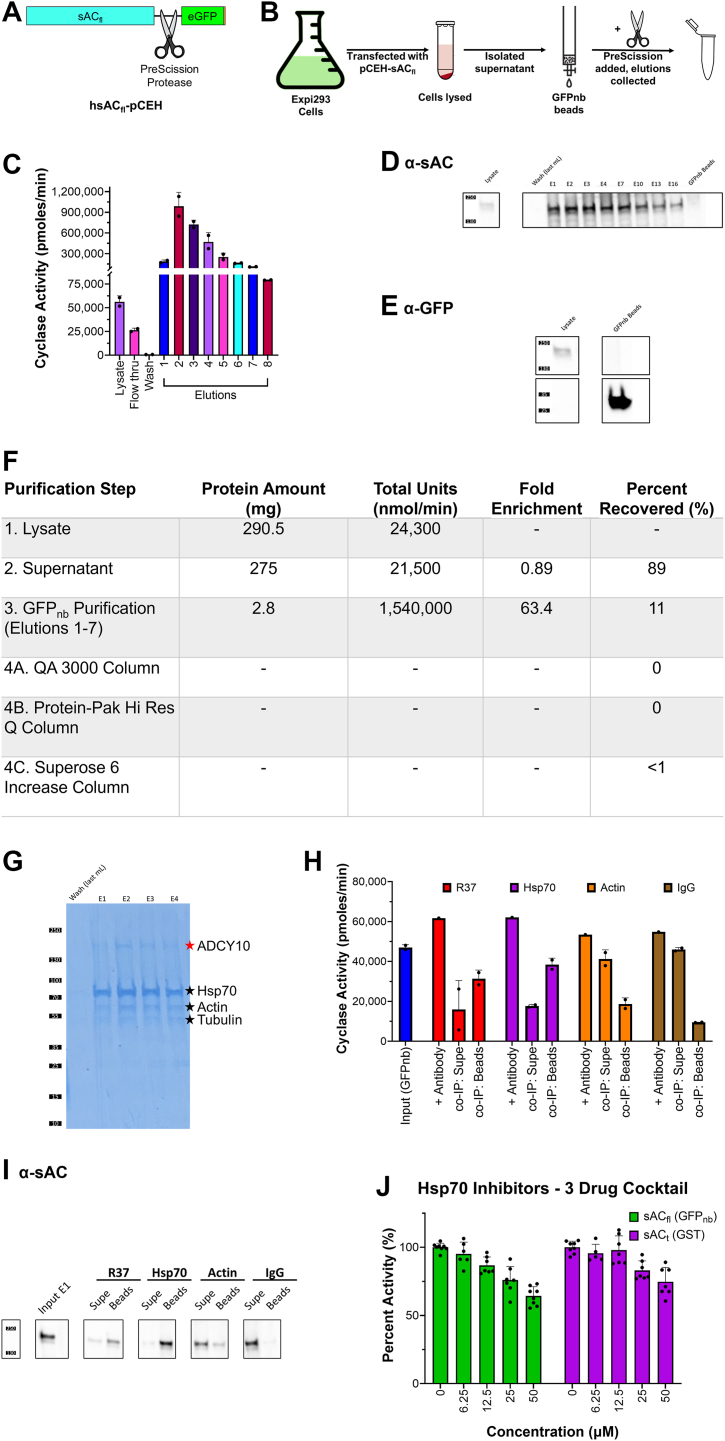
**Purification of sAC_fl_-GPF fusion protein using GFP nanobody purification.***A*, schematic of human sAC_fl_-eGFP fusion protein with His-tagged eGFP C-terminal to sAC_fl_. *B*, schematic of sAC_fl_-eGFP fusion protein purification with GFP nanobody beads and subsequent elution by PreScission protease. *C*, *in vitro* cyclase activity assay, in different fractions from sAC_fl_-eGFP fusion protein GFP nanobody purification, measured in the presence of 2 mM ATP and 10 mM MnCl_2_. Bars represent averages of duplicate determinations with SDs indicated, from a representative experiment which was repeated three independent times. *D* and *E*, Western blots of different fractions from sAC_fl_-eGFP fusion protein GFP nanobody purification probed for sAC (∼187 kDa) and GFP (∼27 kDa), respectively. Elution 2 to 4 have the highest activity and intensity on Western blot for sAC_fl_, 187 kDa. *F*, purification table with percent recovered from each step. Table includes purification steps used to attempt to separate sAC_fl_ from Hsp70 that did not recovery any activity. *G*, Coomassie stain of fractions eluted from GFP nanobody beads mass spectrometry identified the eluting proteins to be sAC_fl_ (187 kDa, *red star*) and Hsp70 (70 kDa), tubulin α/β (55 kDa), and actin (42 kDa) (*black stars*). *H*, cyclase assay of coimmunoprecipitation (co-IP) with various antibodies in the presence of 2 mM ATP and 10 mM MnCl_2_. The antibodies used in this experiment were R37 (*red*, anti-sAC antibody (39), which immunoprecipitates sAC without inhibiting its activity), anti-Hsp70 (*purple*), anti-actin (*orange*), and IgG (negative control, *brown*). Input refers to the eluted fraction used for the co-IP. “+ Antibody” is the corresponding antibody added to the purified fraction, to make sure the antibody did not inhibit cyclase activity. “Co-IP: Supe” and “co-IP: Beads” refers to the co-IP fractions supernatant and protein A/G beads, respectively. Since proteins were not eluted from the beads, the resulting supernatant and beads from the co-IP were tested. Bars represent averages of duplicate determinations with SDs indicated, from a representative experiment which was repeated two independent times. *I*, Western blots of different co-IP fractions probed for sAC (∼187 kDa). sAC_fl_ bands had more activity and were higher intensity in the Western blot on the anti-Hsp70 beads than in the supernatant, meaning it was pulled out of solution by the Hsp70 antibody. *J*, cyclase activity of sAC_fl_-Hsp70 purified complex or purified human sAC_t_ in the presence of excess 10 mM MgCl_2_, 10 mM CaCl_2,_ and 40 mM NaHCO_3_, and the indicated concentration of 3-drug Hsp70 inhibitor cocktail (PES-Cl, YM-01, and VER-155008) on sAC_fl_ (*red*) and sAC_t_ (*blue*). Numbers indicate individual concentration of each drug in the cocktail (*e.g.*, 50 μM represents a cocktail made up of 50 μM PES-Cl, 50 μM YM-01, and 50 μM VER-155008). Bars represent the averages of three independent experiments, assayed in at least duplicate, with SDs indicated. sAC, soluble adenylyl cyclase.

**Supplementary Figure S1:**
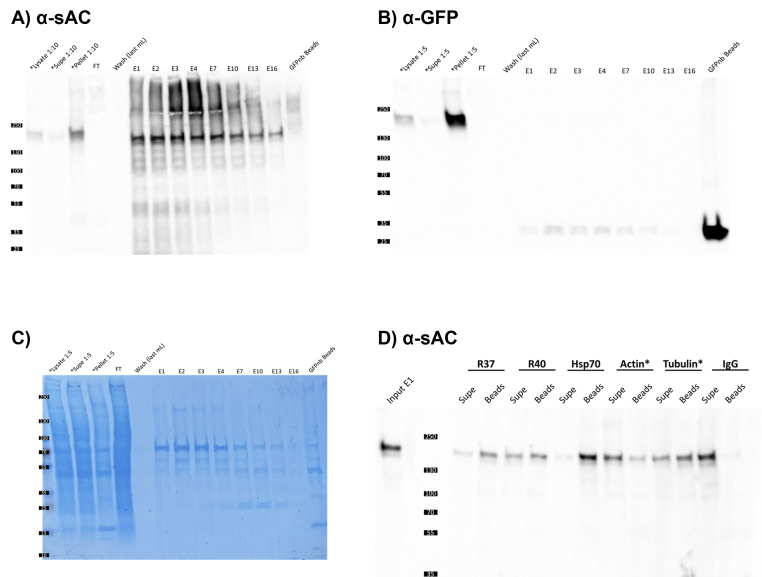
Images of full Western Blots and Coomassie stained SDS/PAGE gels shown in Figure 2. *A* and *B*, full western blots of different fractions from sAC_fl_-eGFP fusion protein GFP nanobody [AMS1] purification probed for sAC (∼187 kDa) and GFP (∼27 kDa), respectively. *C*, full Coomassie stain of purification eluted fractions from pCEH sAC_fl_-GFP transfection. *D*, full western blots of different co-IP fractions probed for sAC (∼187 kDa) and GFP (∼27 kDa) respectively. sAC_fl_ bands had more activity and were higher intensity in the western blot on the beads than in the supernatant, meaning it was pulled out of solution by the Hsp70 antibody. sAC, soluble adenylyl cyclase.

To biochemically characterize sAC_fl_, we expressed sAC_fl_ as a fusion protein with enhanced green fluorescent protein (eGFP) added to the C terminus of human sAC_fl_. We included a PreScission protease site between the sAC_fl_ and eGFP sequences to facilitate purification (Fig. 2*A*). The sAC_fl_-eGFP fusion protein was heterologously expressed in Expi293 cells and purified using GFP nanobody resin (Fig. 2*B*). This method allowed for the overexpression and purification of active sAC_fl_ with considerable levels of recoverable and measurable activity (Fig. 2*C*). Western blotting with antibodies against both sAC and GFP revealed a high molecular weight band of ∼200 kDa corresponding to the sAC_fl_-eGFP fusion protein. A second band at ∼25 kDa detected in the anti-GFP Western corresponded to free eGFP. After purification over the GFP nanobody beads and elution by cleavage with PreScission protease, the eluted fractions no longer contained the ∼200 kDa protein, and the anti-sAC Western blot, but not the anti-GFP Western, detected a protein at ∼187 kDa corresponding to GFP-free sAC_fl_ (Figs. 2, *D*, *E* and S1, *A*, *B*). During purification, there was a ∼64-fold enrichment of sAC cyclase activity (Fig. 2*F*).

### Hsp70 forms an obligatory complex with sAC_fl_

Mass spectrometry (MS) confirmed the identity of the ∼187 kDa protein was GFP-free sAC_fl_. Coomassie staining revealed sAC_fl_ coeluted with other proteins, and subsequent MS identified the three major coeluting proteins as Hsp70, actin, and tubulin (Figs. 2*G* and S1*C*). We attempted to isolate sAC_fl_ from these coeluting proteins using ion exchange and size-exclusion chromatography methods but all attempts proved unsuccessful (Fig. 2*F*); in every case, all activity and any sAC_fl_ protein detectable *via* Western blotting were lost during chromatography. This led us to hypothesize that Hsp70 formed an obligatory complex with sAC_fl_, which upon separation caused sAC_fl_ to precipitate. Consistent with this hypothesize, sAC_fl_ activity (Fig. 2*H*) and sAC_fl_ protein (Figs. 2*I* and S1*D*) coimmunoprecipitated with anti-Hsp70 antibody, but not with anti-actin or IgG antibody.

Hsp70 is a known chaperone protein with ATPase activity whose presence could contribute to stabilizing sAC_fl_ or it could represent a functional binding partner whose activity contributes to sAC_fl_ activity. We addressed this question by taking advantage of various Hsp70 inhibitors, which act *via* distinct mechanisms of action including targeting: the nucleotide-binding domain (VER-155008), the substrate binding domain (PES-Cl), or the interaction interface of Hsp70 and cochaperones (YM-01) (44). As a control, we tested the effects of these inhibitors on purified sAC_t_, which does not contain any contaminating Hsp70 and whose cyclase activity is clearly independent of Hsp70. When added at extremely high concentrations, the cocktail of Hsp70 inhibitors diminished the cyclase activities of both purified sAC_fl_–Hsp70 complex and Hsp70-free purified sAC_t_, indicating these are nonspecific effects (Fig. 2*J*). At lower concentrations (<12.5 μM), there was a slight inhibition of the sAC_fl_–Hsp70 complex, which could reflect destabilization of the complex or Hsp70 utilization of the sAC substrate ATP. We conclude that Hsp70 was playing a chaperone function, and its complex with heterologously expressed human sAC_fl_ facilitated sAC_fl_ expression and purification from Expi293 cells by enhancing stability and/or solubility. Hsp70 is an ATPase, and to avoid complications from the complexed Hsp70 in subsequent biochemical experiments dependent upon precise substrate ATP concentrations, we included a cocktail combining one Hsp70 inhibitor from each class (PES-Cl, YM-01, and VER-155008) at the highest concentration (12.5 μM) where there was no nonspecific inhibition in sAC_t_. Given these results, we proceeded with this partially purified preparation for the experiments described below.

### Knock-in mouse model confirms physiologically expression of sAC_fl_

**Figure 3:**
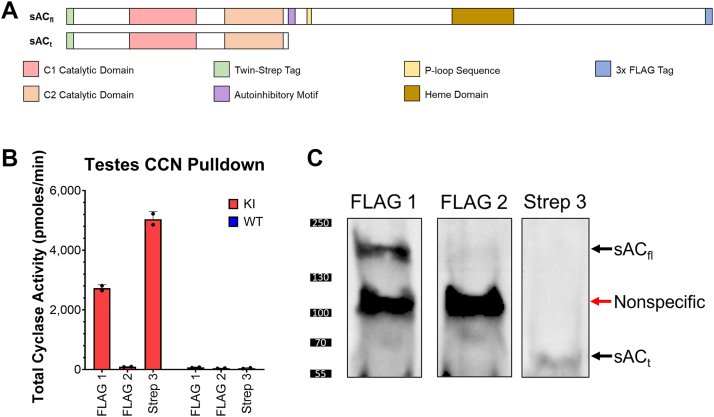
**The generation of a novel knock-in mouse which demonstrates the functional expression of both sAC_fl_ and sAC_t_ in testes.***A*, schematic of different sAC isoforms with catalytic domains, regulatory domains, N-terminal Twin-Strep-tag, and C-terminal 3xFLAG-tag highlighted. *B*, *in vitro* cyclase activity, measured in the presence of 2 mM ATP and 10 mM MnCl_2_, of different fractions from sequential CCN pulldown. Activity on the first FLAG beads corresponds to sAC_fl_ activity while activity on the third pulldown using the Strep beads corresponds to sAC_t_ and any other isoforms not containing a C terminus. Bars represent averages of duplicate determinations with SDs indicated, from a representative experiment which was repeated five independent times. *C*, anti-sAC Western blot of beads from CCN pulldown. sAC_fl_ can be seen in the first FLAG (C terminus) beads at ∼187 kDa and sAC_t_ can be seen in the last Strep (N terminus) beads at ∼53 kDa. The band at ∼115 kDa is a nonspecific band from the FLAG beads. sAC, soluble adenylyl cyclase.

We were concerned that the obligatory complex with the Hsp70 chaperone protein meant that the heterologously expressed human sAC_fl_ was missing a necessary binding partner whose absence could cloud our *in vitro* studies of sAC_fl_ activity. Therefore, we developed a complementary approach to isolate and purify endogenous mouse sAC_fl_. We generated a knock-in (KI) mouse with distinct tags on either end of the *ADCY10* gene; on the N terminus, we knocked in a Twin-Strep-tag; and on the C terminus, we inserted a 3xFLAG-tag (Fig. 3*A*). Starting from testes from this double-tagged KI mouse, we performed sequential pulldowns consisting of a C-terminal (FLAG) pulldown followed by additional C-terminal (FLAG) pulldowns to ensure the first pulldown was comprehensive, and finally an N-terminal (Strep) pulldown (termed CCN pulldowns). The subsequent FLAG pulldowns were repeated until all sAC isoforms with a C terminus were removed to confirm they would not be contaminating the final N-terminal pulldown (Fig. 3*B*).

**Supplementary Figure S2:**
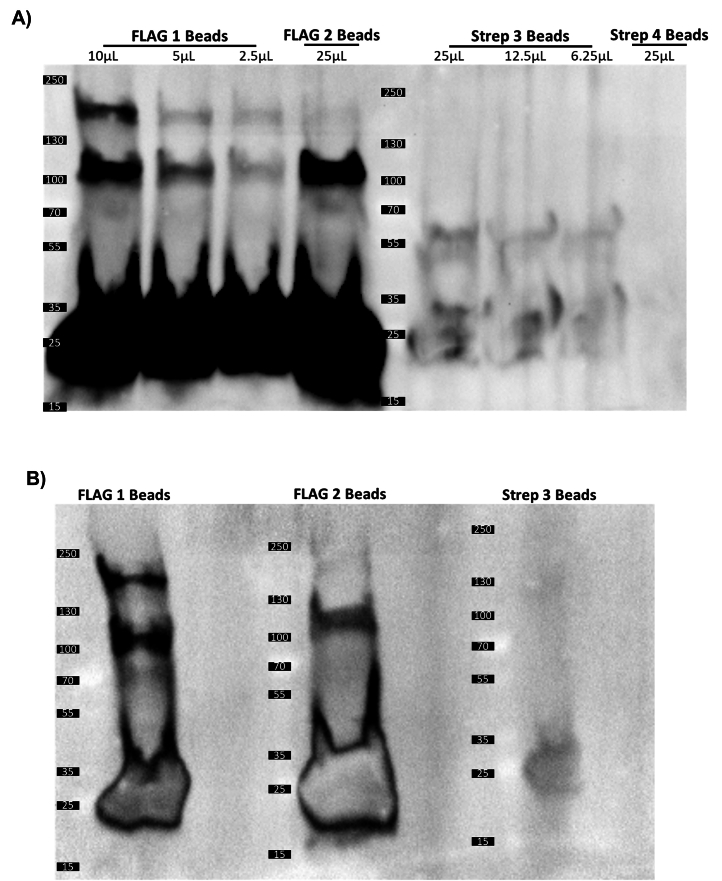
Images of full Western Blots shown in to Figure 3. *A*, full western blots of beads from a CCN pull-down probed for sAC (∼187 kDa). *B*, full western blots of beads from a CCN pull-down probed for FLAG. sAC, soluble adenylyl cyclase.

We detected cyclase activities in both the initial C-terminal and N-terminal pulldowns. As expected, no activity was detected in control pulldowns from WT mice which do not contain the tags on the *ADCY10* gene (Fig. 3*C*). Western blotting confirmed sAC_fl_ presence in the C-terminal pull-down (band at ∼187 kDa; probing for sAC and for FLAG) and sAC_t_ presence in the N-terminal pulldown (band at ∼53 kDa; probing for sAC) (Figs. 3*D* and S2). As seen previously (6, 34, 38), sAC_t_ exhibited higher specific activity (∼3.5-fold) relative to sAC_fl_ (Fig. 2, *C* and *D*). Consistent with our hypothesis that the Hsp70 was acting as a chaperone when we heterologously overexpressed human sAC_fl_ in Expi293 cells, we saw no evidence, either by specific Western blotting or in a MS analysis of the FLAG beads, that mouse Hsp70 was complexed with endogenous mouse sAC_fl_. These data confirm that mouse testis express two alternatively spliced sAC isoforms (*i.e.*, sAC_t_ and sAC_fl_), and the sequential pull-down method from our double KI mouse provides us with a mechanism for purifying and biochemically characterizing both mouse proteins independently.

### Assessing sAC_fl_ biochemistry

After eluting endogenous mouse sAC_fl_ and sAC_t_ from the FLAG-tag and Strep-tag affinity matrices, respectively, we had a suitable source of purified mouse sAC_fl_ and mouse sAC_t_ to directly compare the biochemical profiles of these two naturally occurring sAC isoforms. As mentioned above, the known biochemical properties of sAC are predominantly based upon studies of human heterologously expressed sAC_t_; thus, we include that protein in our comparison. In parallel, to be able to confidently discern whether there are species differences between mouse and human sAC proteins, as well as to assess whether the activity of the heterologously expressed human sAC_fl_ complexed with Hsp70 reflects physiologically relevant sAC_fl_ activity, we also included this protein in our comparison. As stated above, the assays of human sAC_fl_ where substrate ATP has to be precisely controlled, we included a cocktail of Hsp70 inhibitors to avoid complications from the complexed Hsp70. Thus, we performed our *in vitro* cyclase assays using four sAC proteins in parallel; mouse sAC_t_ and sAC_fl_ independently purified from testis, and human sAC_t_ and sAC_fl_ purified following heterologous expression.

**Figure 4:**
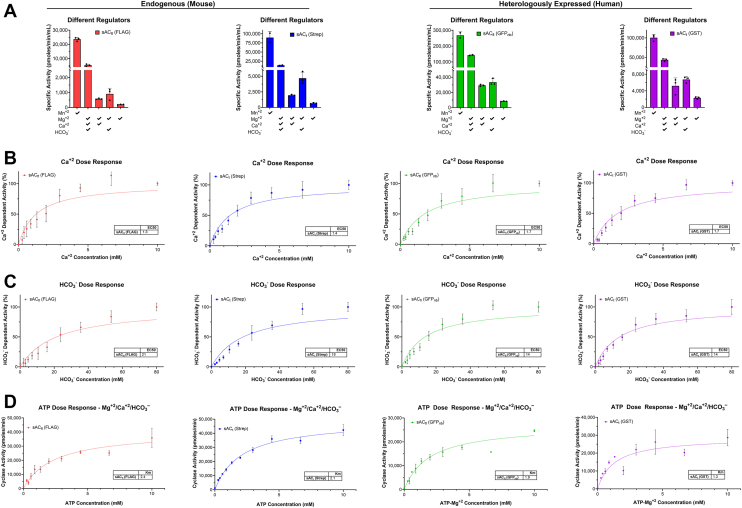
**Known physiologic regulators of sAC_t_ also regulate sAC_f__l_.***A*–*D*, endogeneous mouse sAC: sAC_fl_ (FLAG, *red*) and sAC_t_ (Strep, *blue*) and heterologously expressed human sAC: sAC_fl_ (GFP_nb_, *green*) and sAC_t_ (glutathione-*S*-transferase (GST), *purple*) activity was assayed under different conditions. Unless otherwise indicated reactions activity was assayed in the presence of 2 mM ATP. *A*, different combinations of known sAC physiologic regulators in the presence of excess 10 mM MnCl_2_, 10 mM MgCl_2_, 10 mM CaCl_2_, and/or 40 mM NaHCO_3_. Bars represent averages of at least duplicate determinations with SDs indicated, from a representative experiment which was repeated three independent times. *B*, cyclase activity measured in the presence of the indicated concentration of CaCl_2_ and 10 mM MgCl_2_, and 40 mM NaHCO_3_. Data points represent the averages of three independent experiments, assayed in duplicate, with SDs indicated. *C*, cyclase activity measured in the presence of the indicated concentration of NaHCO_3_ and 10 mM MgCl_2_ and 10 mM CaCl_2_. Data points represent the averages of three independent experiments, assayed in duplicate, with SDs indicated. *D*, cyclase activity measured as a function of substrate [Mg^+2^-ATP] in the presence of 20-40 mM MgCl_2_, 10 mM CaCl_2_, and 40 mM NaHCO_3_. Data points represent averages of duplicate determinations with SDs indicated, from a representative experiment which was repeated at least two independent times. All EC_50_ and K_M_ values are listed in Table 1. sAC, soluble adenylyl cyclase.

Our biochemical characterization of the four proteins consisted of a three-pronged approach exploring: known physiologic regulators of sAC_t_, known pharmacologic inhibitors of sAC_t_, and lastly other potential regulators that might be unique to sAC_fl_. We first assessed how each of the four proteins responded to known physiologic modulators of sAC. While historically, sAC was shown to have its highest *in vitro* activity in the presence of Mn^2+^-ATP (6, 15, 45, 46), the physiological relevant activity of sAC_t_ is stimulated by both Ca^2+^ and HCO_3_^–^ ions and together these have a synergistic effect on cyclase activity. In the presence of different combinations of these various activators, the four purified proteins responded similarly. The highest cyclase specific activity was observed in the presence of Mn^2+^-ATP, and Ca^2+^ and HCO_3_^–^ ions were synergistic in the presence of Mg^2+^-ATP (Fig. 4*A*). Knowing that both sAC_fl_ and sAC_t_ isoforms and both human and mouse proteins respond to the known physiologic activators, we further characterized these four purified proteins by measuring their affinities for substrate and individual regulators (Table 1).

**Table 1:** Summary of biochemical characterization of human and mouse sAC_fl_ and sAC_t_ isoforms

	Endogenous (mouse)		Heterologous (human)	
	sAC_fl_ (FLAG)	sAC_t_ (Strep)	sAC_fl_ (GFP)	sAC_t_ (GST)
Physiologic regulators				
Ca^2+^	1.3 ± 0.15 mM	1.4 ± 0.11 mM	1.7 ± 0.15 mM	1.7 ± 0.16 mM
HCO_3_^–^	21 ± 1.9 mM	19 ± 1.6 mM	14 ± 1.2 mM	14 ± 1.0 mM
ATP	2.2 ± 0.39 mM	2.7 ± 0.25 mM	2.2 ± 0.30 mM	1.8 ± 0.28 mM
Pharmacologic regulators				
TDI- 11861	22.4 ± 9.3 nM	21.1 ± 4.5 nM	5.3 ± 0.9 nM	4.5 ± 0.5 nM


Other potential pegulators	
Pyrophosphate	∼25% inhibition at 1 mM, >90% inhibition at 10 mM
cAMP	∼25% inhibition at 10 mM
UTP	∼50% inhibition at 10 mM
CTP	>50% inhibition at 10 mM
TTP	∼50% inhibition at 10 mM
ITP	∼50% inhibition at 10 mM
Adenosine	>50% inhibition at 10 mM
AMP	No inhibition at 10 mM
ADP	∼75% inhibition at 10 mM
GMP	No inhibition at 10 mM
GDP	∼75% inhibition at 10 mM
GTP	∼50% inhibition at 10 mM

K_M_ value for substrate Mg^2^^+^-ATP in the presence of Mg^2^^+^, Ca^2^^+^, and HCO_3_^-^ and EC_50_ values for Ca^2^^+^, HCO_3_^-^, products (pyrophosphate, and cAMP), sAC inhibitor (TDI-11861), and all nucleotides tested are listed.

While the EC_50_ values for Ca^2+^ were very similar, ∼1.3 to 1.7 mM, across the different isoforms and different species for sAC (Fig. 4*B*), the EC_50_ values for HCO_3_^–^ were not consistent between the four proteins. Within each species the sAC_fl_ and sAC_t_ isoforms did not differ, but we did observe a potential species difference; the EC_50_ for HCO_3_^–^ was ∼20 mM for both human sAC isoforms and ∼14 mM for both mouse sAC isoforms (Fig. 4*C*). These differences may reflect the presence of distinct affinity tags on the mouse proteins compared to the human proteins, or the fact that the mouse proteins were purified from endogenous tissue compared to expression in tissue culture cells for the human proteins. We next compared how all four proteins responded to changing levels in substrate. Because the K_M_ of sAC’s substrate, ATP, is ∼2 mM, which is close to the levels found in most cells, sAC activity is thought to be physiologically regulated by fluctuations in cellular ATP levels (16). In the presence of its known physiological regulators (*i.e.*, Mg^2+^, Ca^2+^, and HCO_3_^–^ ions), mouse and human sAC_fl_ and sAC_t_ exhibited similar K_M_ of 1 to 2 mM (Fig. 4*D*). Independent of the potentially species difference in affinity for bicarbonate, the observation that sAC_fl_ and sAC_t_ proteins share similar affinities for substrate and their physiological regulators is not surprising because the binding sites of substrate ATP and these regulators were structurally demonstrated to be in the active site, which is formed at the interface between the C1 and C2 catalytic domains and shared between sAC_fl_ and sAC_t_ (42).

**Figure 5:**
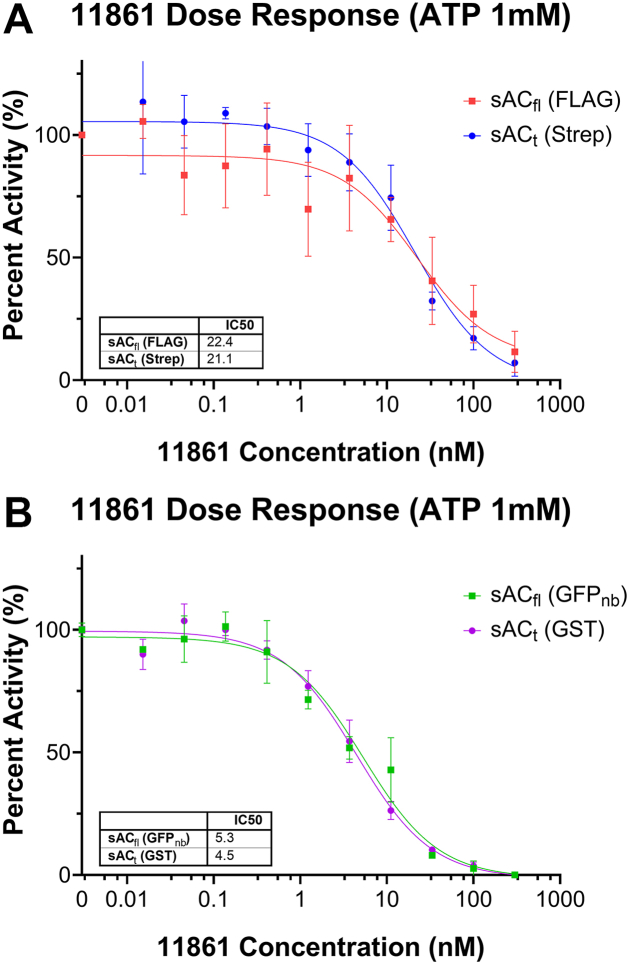
**Known pharmacologic regulators of sAC have similar effects on sAC_fl_ and sAC_t_.***A*, cyclase activities of TDI-11861 on endogenous mouse sAC_fl_ (*red*) and sAC_t_ (*blue*) measured in the presence of 10 mM MgCl_2_, 10 mM CaCl_2_, and 40 mM NaHCO_3_ and the indicated concentration of 11861. *B*, cyclase activities of heterologously expressed human sAC_fl_ (*green*) and sAC_t_ (*purple*) measured in the presence of 10 mM MgCl_2_, 10 mM CaCl_2_, and 40 mM NaHCO_3_ and the indicated concentration of 11861. IC_50_ values are listed in Table 1. Data points represent the averages of three independent experiments with standard deviations indicated. sAC, soluble adenylyl cyclase.

We next compared responsiveness to pharmacologic regulators of sAC. Currently, there are multiple inhibitors specific for sAC available, the most potent and recently discovered being TDI-11861 (47). Because TDI-11861 is structurally shown to bind to the regulatory site and extend into the active site which is shared between sAC_fl_ and sAC_t_, it is not surprising that its IC_50_ values were similar when targeting either isoform within each species. However, as with the HCO_3_^–^ EC_50_ value, there was a species difference in the IC_50_ values between human, which was ∼5 nM, and mouse, which was ∼21 nM (Fig. 5). TDI-11861 is a competitive antagonist with HCO_3_^–^ and binds in the bicarbonate binding site of sAC. The bicarbonate binding site is shared between sAC_fl_ and sAC_t_, which may help explain why the IC_50_ values are similar within species. However, the difference in IC_50_ values between human and mouse may be related to the differences seen in EC_50_ values of HCO_3_^–^.

**Supplementary Figure S3:**
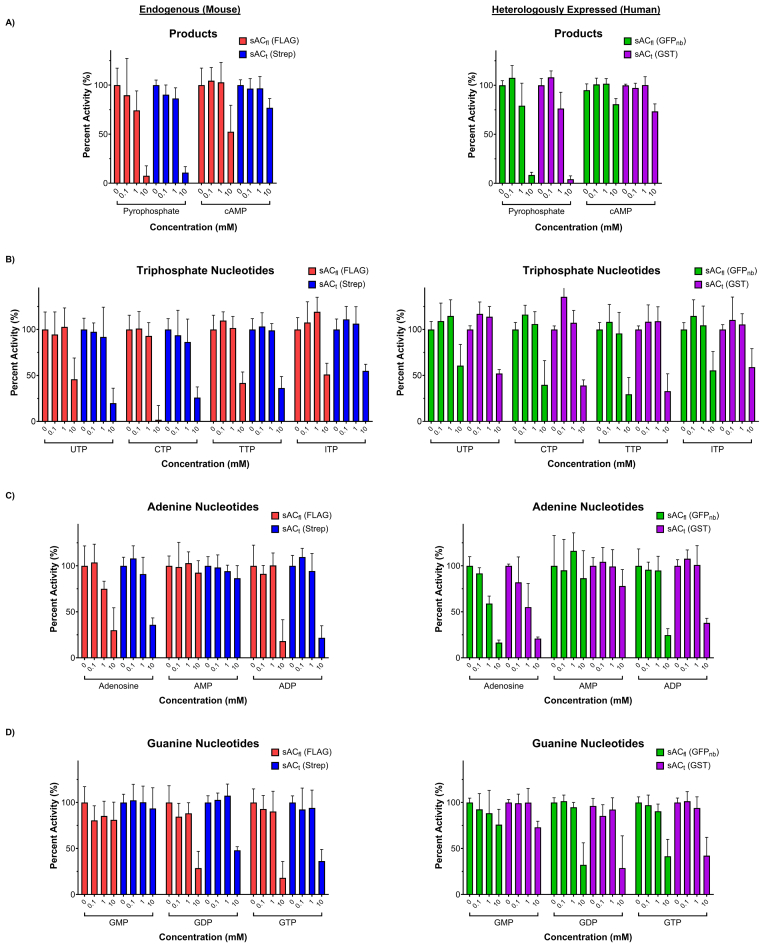
Adenylyl cyclase activities of other regulators listed in Table 1. sAC activities measured in the presence of different small molecules. Endogenous sAC: sAC_fl_ (FLAG, *red*) and sAC_t_ (Strep, *blue*) and heterologously expressed sAC: sAC_fl_ (GFP_nb_, *green*) and sAC_t_ (GST, *purple*) activities were measured in the presence of 2mM ATP, excess 10mM MgCl_2_, 10mM CaCl_2_, and 40mM NaHCO_3_. Reactions were performed in the presence of indicated concentrations of (A) products: pyrophosphate and cAMP; (B) various other triphosphate nucleotides including CTT, UTP, ITP, and TTP; (C) various other adenosine-based nucleotides including adenosine, ADP, and AMP; and; (D) various other guanine-based nucleotides including GMP, GDP, and GTP. *Bars* represent the averages of three independent experiments, assayed in duplicate, with standard deviations indicated. sAC, soluble adenylyl cyclase.

**Supplementary Figure S4:**
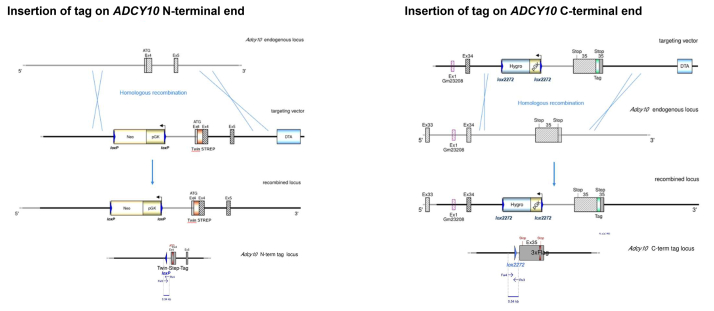
**Schematic of knock-in (KI) mouse generation, with Twin-Strep-tag inserted on *ADCY10* N-terminal end and 3xFLAG-tag inserted on *ADCY10* C-terminal end**.

The observation that sAC_fl_ or sAC_t_ within a species responded similarly to known physiological and pharmacological regulators is not surprising since each of these modulators bind to and function *via* the shared C1 and C2 catalytic domains. We next turned our attention towards potential modulators of sAC_fl_ which might show differential regulation of sAC_fl_ *versus* sAC_t_. We first tested the products of the cyclase reaction, pyrophosphate and cAMP. All four sAC proteins responded similarly, pyrophosphate had slight inhibition at 1 mM and fully inhibited cyclase activity at concentrations >10 mM; while cAMP only had minimal inhibition at concentrations >10 mM (Fig. S4*A*). As mentioned above, sAC_fl_ sequence contains putative C-terminal regulatory domains absent in sAC_t_, including a predicted STAND domain containing a P-loop (35). P-loops are known to bind nucleotides to regulate enzymatic activity, so we tested whether different nucleotides were capable of regulating the cyclase activity of sAC_fl_. We tested five NTPs: CTP, UTP, TTP, ITP, and GTP; adenosine; AMP; ADP; GMP; and GDP (Fig. S3, *B*–*D*). With all nucleotides tested, there were no distinguishing effects between the four sAC proteins tested; that is, we observed no isoform or species-specific differences. The NTPs exhibited some inhibition when added at 10 mM, which is 10-fold above the concentration of substrate ATP. Adenosine, ADP, and GDP also showed some inhibition in all four proteins at 10 mM, while there was no inhibition at concentrations up to 10 mM for AMP and GMP (Table 1). Inhibition of sAC_t_ or sAC_fl_ isoforms by 10 mM NTPs is consistent with previous results revealing that sAC is able to bind GTP but in a nonproductive manner (42, 48). Additionally, sAC was also postulated to be responsible for cyclization of noncanonical NTPs into the respective cyclic nucleotides (49, 50). UTP and CTP were thought to be substrates for sAC to generate cUMP and cCMP, respectively. However, because UTP and CTP only showed the potential for competitive inhibition of 1 mM ATP when included at supraphysiological levels, use of these nucleotides as substrates seems unlikely. As a whole, the nucleotide data suggest that the other nucleotides tested do not specifically regulate sAC_fl_, and nor are they substrates for sAC; they only act as competitive antagonists at supraphysiological levels.

## Discussion

In this article, we developed a new method to purify heterologously expressed human sAC_fl_, and we describe a novel genetic mouse model which leverages unique N-terminal and C-terminal tags allowing separation of endogenous sAC_fl_ and sAC_t_. Using this novel mouse model, we confirmed that both sAC_fl_ and sAC_t_ are physiologically expressed in murine testes. Having these purified proteins permitted in depth biochemically characterization and comparison of separated mouse and human sAC_fl_ and sAC_t_ isoforms.

Previous attempts to purify sAC_fl_, either through endogenous pulldowns *via* anti-sAC antibodies or overexpression and purification using a His-tag, proved unsuccessful, most often because *in vitro* activity would remain in the insoluble fraction. Here, we leveraged a sAC_fl_-eGFP fusion protein for heterologous expression of sAC_fl_ which resulted in increased solubility of the fusion protein. Expressing the sAC_fl_-eGFP fusion protein also provided an ability to monitor transfection efficiency through GFP fluorescence, ease of purification *via* affinity chromatography, and the ability to cleave the eGFP with PreScission protease leaving native sAC_fl_. In this heterologous expression system, human sAC_fl_-eGFP, as well as the cleaved sAC_fl_, formed an obligatory complex with Hsp70. Because the endogenous mouse sAC_fl_ protein was not bound to Hsp70, we concluded that Hsp70 facilitates sAC_fl_ expression and enhances stability and/or solubility during overexpression in Expi293 cells. This implies that an endogenous protein in male germ cells facilitates expression and/or stability of sAC_fl_ in nature. Previous research suggests, the sperm-specific sodium/proton exchanger SLC9C1 (sNHE), in concert with its binding partner TMEM217, are obligatory binding partners contributing to the stability of sAC_fl_ in sperm (51, 52). It is tempting to hypothesize that because this essential protein–protein interaction between sNHE/TMEM217 and heterologously expressed sAC_fl_ is missing in the overexpression system, the chaperone protein Hsp70 stabilizes sAC_fl_ in its absence.

Direct comparison of the biochemical profiles of human and mouse sAC isoforms confirmed the previously identified difference in specific activities between sAC_fl_ and sAC_t_ (6, 34, 38); using these more highly purified preparations, we observed a ∼3-fold higher specific activity of sAC_t_ relative to sAC_fl_. Because the lower specific activity of sAC_fl_ was found to be dependent upon inclusion of sequences resembling a nucleotide binding P-loop, we tested whether inclusion of various nucleotides might show sAC_fl_-specific stimulation. However, with these purified preparations, which may lack a sAC_fl_ regulatory binding partner or some unknown regulatory metabolite, we did not identify a sAC_fl_-specific regulator.

In the end, we did not identify any discernible differences between sAC_fl_ and sAC_t_ isoform activities in response to physiological or putative regulators, presumably because these isoforms share the same catalytic domains. We did observe a reproducible difference between mouse and human sAC in their affinities for the physiological regulator HCO_3_^–^. Interestingly, previous work provides a precedent for species differences in EC_50_’s for HCO_3_^–^ (13, 15, 53), but in none of those cases were two mammalian sAC proteins from different species compared head-to-head as performed here. Structural modeling of mouse sAC in comparison to the human sAC structure does not suggest a mechanism for the observed difference in HCO_3_^–^ affinity, and it remains an open question whether the observed difference represents a biological difference or reflect a difference between comparing endogenously expressed mouse proteins with heterologously expressed human proteins.

sAC plays an essential role in male fertility, specifically regulating motility and capacitation of sperm (22, 23, 24, 26). It is unknown which sAC isoform is involved in each of these sperm specific processes; the work presented here, separately purifying and characterizing each isoform, represents a first step to better understand the distinct biochemical profiles and specific involvement in sAC-dependent processes of each individual isoform. Currently, sAC-specific inhibitors are being developed as novel, on-demand, nonhormonal male and topical vaginally delivered female contraceptives (25, 31, 32). As expected, these catalytic domain inhibitors do not display isoform selectivity. This lack of isoform selectivity may be important for sAC inhibitor contraceptives because it may prove necessary to inhibit all sAC isoforms in sperm, but there may also be a downside to pan sAC inhibitors. Because sAC is not exclusively expressed in male germ cells, pan-sAC inhibitors may elicit adverse effects outside the germ line. On the other hand, should future studies identify essential roles for an individual sAC isoform, it may be possible to develop novel pharmacologic agents selectively targeting the isoform of interest.

## Experimental procedures

### Materials

All reagents including other nucleotides (adenosine, AMP, ADP, GMP, GDP, GTP, CTP, TTP, ITP, UTP), HSP70 Inhibitors (VER-155008, HS-72, SW02, YM-1, Pifithrin-μ, PES-Cl, Myricetin, Quercetin), and anti-FLAG [M2] magnetic beads were all purchased from Sigma-Aldrich.

Fetal bovine serum, Expi293 cells, Expi293 Expression Medium, ExpiFectamine 293 Transfection Kit, Opti-MEM Medium, NuPAGE LDS Sample Buffer (4X), Pierce Silver Stain Kit, SuperSignal West Atto Ultimate Sensitivity Substrate, SuperSignal West Femto Maximum Sensitivity Substrate were purchased from Thermo Fisher Scientific.

Mini-PROTEAN TGX Precast Protein 4 to 15% gels (15-well/15 μl and 10-well/50 μl) were purchased from Bio-Rad. [α-^32^P]-ATP and [2,8-^3^H]-cAMP were both purchased from Revvity. Protease Inhibitor Cocktail Set III was purchased from Millipore.

Single items purchased were: Mag-Strep Strep-Tactin XT beads from IBA Life Sciences; N-dodecyl-β-D-maltoside (DDM) from Anatrace; Protein-Pak Hi Res Q 5 μm 4.6 x 100 mm from Waters; Superose 6 Increase 10/300 GL Chromatography Column and Gel Filtration HMW Calibration Kit from Cytiva.

### *In vitro* AC activity assay

All *in vitro* AC activity assays utilized the classical “two-column” method, developed by Salomon (54), where conversion of [α-^32^P]-ATP to [^32^P]-cAMP is quantified by sequential Dowex and Alumina chromatography resins to purify the generated [^32^P]-cAMP. Cyclase assays were performed in 100 μl of total reaction volume using 25 to 600 ng of sAC, 50 to 150 mM of Tris–HCl (pH 7.5), 3 mM of DTT, bovine serum albumin 0.03%, substrate [α-^32^P]-ATP, and either MnCl_2_, or MgCl_2_, and/or CaCl_2_, and/or NaHCO_3_ concentrations as indicated (14). Reactions measuring the cyclase activity of cell lysates, organ lysate, KI mouse pulldowns, or sAC_fl_ pre-GFP nanobody purification fractions also contained DNase I 0.1 mg/ml, an ATP regeneration system (CP 20 mM, CPK 100 U/ml), cAMP 1 mM, and phosphodiesterase inhibitors [3-isobutyl-1-methylxanthine 500 μM and dipyridamole 30 μM]. Reactions were initiated by the addition of 10,000,000 to 1,000,000 counts/min of [α-^32^P]-ATP, incubated for 30 min at 30 °C, quenched by adding 200 μl of SDS 2%, and cAMP was quantified following sequential Dowex and Alumina chromatography. An internal standard of ∼10,000 counts/min of [^3^H]-cAMP was added to each reaction. To determine specific activities of endogenous mouse proteins, sAC_fl_ and sAC_t_ were quantitated by Western blot. For ATP kinetics, representative experiments are shown with SEM determined from normalized data.

### Western blot

Protein extracts were subjected to SDS-PAGE and transferred to polyvinylidene difluoride (Thermo Fisher Scientific) at 100 V for 90 min. polyvinylidene difluoride membranes were blocked with 5% skim milk in Tris-buffered saline containing 0.1% Tween-20 (TBS-T) for 1 h. Primary antibodies diluted in TBS-T were as follows: 1/3000 for R21 (anti-sAC, (11)), 1/5000 for anti-GFP (Invitrogen, MA5-15256), 1/3000 for anti-FLAG (Sigma-Aldrich, F1804), 1/2000 for anti-Twin-Strep (IBA Lifesciences), and 1/4000 anti-Hsp70 (Bio-Techne, MAB1663). Secondary antibodies, anti-mouse (Cell Signaling, 7076), or anti-rabbit (Cell Signaling, 7074) were diluted 1/15,000 in TBS-T and the membranes analyzed using an enhanced chemiluminescence detection kit (SuperSignal West Femto Maximum Sensitivity Substrate or SuperSignal West Atto Ultimate Sensitivity Substrate, Thermo Fisher Scientific). Image Lab (Bio-Rad; www.bio-rad.com/en-us/product/image-lab-software) was used for densitometric analysis of Western blots.

When needed, membranes were stripped with 20 ml of Strip-It Buffer (Advansta) and incubated for 5 to 10 min at room temperature. After incubation, membranes were reblocked in 5% skim milk in TBS-T for 1 h and reprobed.

### Expression of sAC_fl_ in Expi293 cells

Human sAC_fl_ coding sequence was cloned into pNVH to generate an N-terminal venus protein fusion protein and pCEH to generate a C-terminal eGFP fusion protein (kind gift of Bryce Delgado and Stephen Long, MSKCC). In both fusion proteins a His-tag is present on the venus or eGFP. Nucleotide sequences of inserted fusion proteins were confirmed. Fusion proteins were expressed in Expi293 cells (ExpiFectamine 293 Transfection Kit, Gibco), harvested after 2 days, and purified as described (55, 56). Both N-terminal and C-terminal fusion proteins were evaluated for expression levels and protein stability, and the C-terminal fusion was found to express better.

### sAC_fl_ protein purification

All steps were performed at 4 °C unless otherwise noted. Cell pellets of transfected Expi293 cells were resuspended in 100 ml of lysis buffer per 1 L of pellet [Tris–HCl 100 mM (pH 7.5), NaCl 150 mM, DTT 1 mM, DNase I 0.1 mg/ml, EDTA 1 mM, EGTA 1 mM, and a 1:250 dilution of Protease Inhibitor Cocktail Set III (Millipore), DDM 1%] and lysed by dounce homogenization then sonication (15% power, 10 s on and 10 s off, 10 min in total; Branson SFX 250). DDM was added to the crude cell lysate and rotated on an orbital rotator for 20 min then pelleted by centrifugation (20,000*g* for 30 min). The supernatant was bound to two batches of GFP nanobody resin, the first round was 2 ml of resin per 1 L of cell pellet followed by a second 1.5 ml of resin per 1 L of cell pellet batch (55, 57). Both batches were pooled, transferred to a gravity column, and washed with 90 ml of wash buffer [Tris–HCl 100 mM (pH 7.5), NaCl 300 mM, DTT 1 mM, EDTA 1 mM, and EGTA 1 mM]. sAC_fl_ was eluted from the GFP nanobody affinity column by proteolysis with 0.4 mg of PreScission protease, 3 h of incubation in buffer supplemented with 1 mM DTT.

Eluted fractions from the GFP nanobody beads were diluted to decrease NaCl concentration and pH adjusted for anion exchange columns or concentrated (Amicon Ultra-4 10 kDa centrifugal filters, Millipore) for size exclusion column. Protein-Pak Hi Res Q HPLC (Waters, 5 μm, 4.6 × 100 mm) column was run in 20 mM Tris, pH 8.0, with a linear gradient from 0 to 500 mM NaCl at a flow rate of 0.5 ml/min and psi <2000. Superose 6 Increase 10/300 GL, (Cytiva, 10 × 300–310 mm) column was run in PBS without Ca^2+^ and Mg^2+^ (Cytiva) at a flow rate of 0.5 ml/min and psi <250. QA 3000 column was run in 20 mM Tris, pH 7.5, from 0 to 500 mM NaCl at a flow rate of 0.5 ml/min and psi <4000. When needed, fractions were concentrated in a speed vacuum (18 h with no heat, pressure level of 0.1 torr, rate of 70 torr/min) and then resuspended in HPLC-H_2_O prior to subsequent experiments.

### MS identification of copurifying proteins

Desalted peptides from the GFP purification elutions were separated using NanoAcquity UPLC system (Waters) equipped with a Acquity UPLC BEH C18 column (Waters, 75 μm × 250 mm) and a 180 μm × 20 mm trap column (Waters). Columns were run in 0.1% formic acid with a linear gradient from 1 to 90% acetonitrile at a flow rate of 300 nl/min. Duplicates from a single GFP purification were analyzed.

MS data were acquired on a Q Exactive mass spectrometer (Thermo Fisher Scientific). Full MS1 scans were acquired at a resolution of 70,000 with an automatic gain control (AGC) target of 1 × 10^6^, a maximum injection time of 50 msec, and scan range of 380 to 1600 *m/z*. Data were collected in data-dependent acquisition mode, targeting the top 10 most intense precursor ions for higher energy collisional dissociation fragmentation performed at a normalized collision energy of 27% with an AGC target of 2.0 × 10^4^, an isolation window of 1.5 *m/z*, and a dynamic exclusion of 15 s. Tandem mass spectrometry spectra were acquired at a resolution of 17,500. For database search, raw files were processed using Mascot and searched for protein identification against the SwissProt protein database for human (downloaded January 7th, 2023). Carbamidomethylation of C was set as a fixed modification and the following variable modifications allowed: oxidation (M), N-terminal protein acetylation, deamidation (N and Q), and phosphorylation (S, T, and Y). Search parameters specified an MS tolerance of 10 ppm, an tandem mass spectrometry tolerance at 0.080 Da and full trypsin digestion, allowing for up to two missed cleavages. False discovery rate was restricted to 1% in both protein and peptide level. Protein intensities were obtained using Scaffold (5.3.4).

### IPs of partially purified hsAC_fl_

All steps were performed at 4 °C unless otherwise noted. Semipurified fractions from GFP nanobody purification were incubated in equal amounts with respective antibody, 6 μg of each, for 18 h overnight with mixing on a nutator in IP buffer [Tris–HCl 100 mM (pH 7.5), NaCl 300 mM, DTT 1 mM]. The antibodies used were R37 (anti-sAC antibody (39), which immunoprecipitates sAC without inhibiting its activity), anti-Hsp70, anti-actin, and IgG (negative control). To bind the antibody–antigen complex, 10 μl of Pierce Protein A/G Magnetic Beads (Thermo Fisher Scientific) were incubated with the complex for 2 h at room temperature while mixing on a nutator. Prior to being added to the supernatant, beads were washed three times with five bead volumes each of IP buffer. After incubation, beads were collected on the magnetic stand and resulting flow-through was collect and assayed directly (*i.e.*, *via* cyclase assay or Western blot).

### KI mouse generation

A strain of mice harboring both an N-terminal Twin-Strep-tag and a C-terminal 3xFLAG-tag at the *ADCY10* locus was generated by genOway by KI insertion of an N-terminal Twin-Strep-tag and a C-terminal 3xFlag-tag as shown in Figure S4. Targeting vectors were inserted into ES cells sequentially and both loci confirmed by complete nucleotide sequencing. Neo cassettes were excised from hybrid mice *via* breeding with C57BL/6N Cre-deleter mice to generate heterozygous mice carrying the double KI allele (Fig. S4). Animal experiments were approved by Weill Cornell Medicine’s Institutional Animal Care and Use Committee.

### 3xFLAG-tag and Twin-Strep-tag affinity pulldowns

All steps were performed at 4 °C unless otherwise noted. Mouse testes were dissected from WT and homozygous double KI (3xFLAG- and Twin-Strep-tag) mice, resuspended in 1 μl/mg of lysis buffer [Tris–HCl 100 mM (pH 7.5), NaCl 150 mM, DNase I 0.1 mg/ml, EDTA 1 mM, EGTA 1 mM, a 1:250 dilution of Protease Inhibitor Cocktail Set III (Millipore), DDM 1%], and lysed by Dounce homogenization. DDM was added to the crude organ lysate and rotated on an orbital rotator for 30 min and then pelleted by centrifugation (20,000*g* at 4 °C for 30 min). Supernatant was isolated and used in subsequent pull-down experiments.

For Twin-Strep-tag (N terminus) pulldowns, 5 μl (100 μl of 5% slurry) of Mag-Strep Strep-Tactin XT beads (IBA Lifesciences) were used per 200 μl of supernatant. For FLAG-tag (C terminus) pulldowns, 12.5 μl (25 μl of 50% slurry) of anti-FLAG M2 antibody magnetic beads (Millipore) were used per 200 μl of supernatant. For sequential pulldowns where the flow-through was decreased by over 10%, the amount of beads was decreased by the same percentage. Prior to being added to the supernatant, beads were washed three times with five bead volumes each of wash buffer [Tris–HCl 100 mM (pH 7.5), NaCl 300 mM, EDTA 1 mM, EGTA 1 mM]. Beads were incubated at room temperature for 2 h with extract while mixing on a nutator. After incubation, beads were collected on the magnetic stand and resulting flow-through was collect and saved for subsequent affinity pull-downs experiments or analysis. Beads were then washed three times (20 bead volumes total) with wash buffer with detergent [Tris–HCl 100 mM (pH 7.5), NaCl 300 mM, EDTA 1 mM, EGTA 1 mM, DDM 0.05%] and assayed directly (*i.e.*, *via* cyclase assay or Western blot) or stored for subsequent elution.

Different elution buffers were used for the anti-FLAG M2 antibody magnetic beads and Mag-Strep Strep-Tactin XT beads: 3xFLAG peptide elution buffer [Tris–HCl 100 mM (pH 7.5), NaCl 150 mM, 3xFLAG peptide 150 ng/ml, bovine serum albumin 0.1%] and Twin-Strep elution buffer [Tris–HCl 100 mM (pH 8.0), NaCl 150 mM, Biotin 50 mM, bovine serum albumin 0.1%]. Beads were collected with a magnetic stand to remove wash buffer then two bead volumes of respective elution buffer was added and incubated at room temperature for 30 min with mixing on a nutator. After incubation, beads were collected on the magnetic stand and resulting elution collected. Elution step was repeated until a total of 10 bead volumes was used.

### Statistical analysis

Plotting data, curve fitting, and statistical analyses were performed using the GraphPad Prism software (GraphPad: www.graphpad.com, version 10.2.3). All data are shown as the mean ± SD

## Data availability

All data generated and analyzed in this study are included in the article and its supplementary information files. Any additional data related to this study are available upon request from the corresponding author.

## Supporting information

This article contains supporting information.

## Conflicts of interest

L. R. L. and J. B. are coinventors of a panel of *in vivo*, validated sAC inhibitors and are cofounders of Sacyl Pharmaceuticals Inc., which licensed the sAC inhibitors for development into contraceptives. The other authors declare that they have no conflicts of interest with the contents of this article.
