# Supplementary material for: Biochemical characterization of the full-length isoform of soluble adenylyl cyclase
Source: J Biol Chem. 2025 Nov 5;301(12):110889. doi: 10.1016/j.jbc.2025.110889 (PMC12802110; doi:10.1016/j.jbc.2025.110889)
Supplement: Supplementary Figure Legends [file mmc1.docx]

**Supplementary Figure 1**

**A and B)** Full Western blots of different fractions from sAC_fl_-eGFP fusion protein GFP nanobody purification probed for sAC (~187 kDa) and GFP (~27 kDa) respectively. **C)** Full Coomassie stain of purification eluted fractions from pCEH sAC_fl_-GFP transfection. **D)** Full Western blots of different co-IP fractions probed for sAC (187 kDa) and GFP (27 kDa) respectively. sAC_fl_ bands had more activity and were higher intensity in the Western blot on the beads than in the supernatant, meaning it was pulled out of solution by the Hsp70 antibody.

**Supplementary Figure 2**

**A)** Full Western blots of beads from a CCN pull-down probed for sAC (~187 kDa). **B)** Full Western blots of beads from a CCN pull-down probed for FLAG.

**Supplemental Figure 3**

**A-D)** sAC activities measured in the presence of different small molecules. Endogenous sAC: sAC_fl_ (FLAG, red) and sAC_t_ (Strep, blue) and heterologously expressed sAC: sAC_fl_ (GFP, green) and sAC_t_ (GST, purple) activities were measured in the presence of 2mM ATP, excess 10mM MgCl_2_, 10mM CaCl_2_, and 40mM NaHCO_3_. Reactions were performed in the presence of indicated concentrations of **A)** products: pyrophosphate and cAMP; **B)** various other triphosphate nucleotides including: CTT, UTP, ITP, and TTP; **C)** various other adenosine-based nucleotides including: Adenosine, ADP, and AMP; and **D)** various other guanine-based nucleotides including: GMP, GDP, and GTP. Bars represent the averages of 3 independent experiments, assayed in duplicate, with standard deviations indicated.

**Supplementary Figure 4**

Schematic of Knock-In (KI) mouse generation, with Twin-Strep-tag inserted on *ADCY10* N-terminal end and 3xFLAG-tag inserted on *ADCY10* C-terminal end.
